# The Case against Antibiotics and for Anti-Virulence Therapeutics

**DOI:** 10.3390/microorganisms9102049

**Published:** 2021-09-28

**Authors:** Julia A. Hotinger, Seth T. Morris, Aaron E. May

**Affiliations:** Department of Medicinal Chemistry, School of Pharmacy, Virginia Commonwealth University, Richmond, VA 23219, USA; hotingerja@vcu.edu (J.A.H.); morriss3@vcu.edu (S.T.M.)

**Keywords:** antibiotics, anti-virulence, resistance, type III secretion system, quorum sensing, liposomes, commensal bacteria

## Abstract

Although antibiotics have been indispensable in the advancement of modern medicine, there are downsides to their use. Growing resistance to broad-spectrum antibiotics is leading to an epidemic of infections untreatable by first-line therapies. Resistance is exacerbated by antibiotics used as growth factors in livestock, over-prescribing by doctors, and poor treatment adherence by patients. This generates populations of resistant bacteria that can then spread resistance genes horizontally to other bacterial species, including commensals. Furthermore, even when antibiotics are used appropriately, they harm commensal bacteria leading to increased secondary infection risk. Effective antibiotic treatment can induce bacterial survival tactics, such as toxin release and increasing resistance gene transfer. These problems highlight the need for new approaches to treating bacterial infection. Current solutions include combination therapies, narrow-spectrum therapeutics, and antibiotic stewardship programs. These mediate the issues but do not address their root cause. One emerging solution to these problems is anti-virulence treatment: preventing bacterial pathogenesis instead of using bactericidal agents. In this review, we discuss select examples of potential anti-virulence targets and strategies that could be developed into bacterial infection treatments: the bacterial type III secretion system, quorum sensing, and liposomes.

## 1. Introduction

Before the serendipitous discovery of penicillin in 1929, the three leading causes of death in the United States were all infectious diseases: influenza, pneumonia, and tuberculosis [1,2]. Pathogenic bacteria, although no longer leading causes of death due to antibiotics, continue to be a global health threat, with a billion infections worldwide and nearly 15 million deaths every year [3,4,5]. The prevalence of antibiotic-resistant bacterial pathogens is a serious and growing problem. The CDC estimates that the rate of antibiotic-resistant infections in the United States has increased by 40% from 2014 to 2019 [6]. Moreover, the observance of resistance to each new antibiotic is never far behind the commercial release of the drug [6]. This contributes to a slowing of novel antibiotics coming to market, with 78% of major drug companies shown to decrease or stop antibiotic research between 1990 and 2019 [7].

Deleterious effects of antibiotic use, other than resistance, have become more well understood over time. Antibiotics can destroy commensal bacterial colonies, leading to an increased risk of infection and other health complications [8,9,10]. They may also induce bacterial SOS tactics, which can include toxin release and increased transfer of resistance genes [11,12]. Although there are mediation methods, such as combination therapies, narrow-spectrum antibiotics, and stewardship programs, the root cause of these issues is that antibiotics directly kill bacteria. An alternative to antibiotic treatments could be anti-virulence therapies. Anti-virulence therapies target the pathogenic mechanisms of bacteria rather than killing bacteria outright. In this review, we present lesser-known evidence of the detrimental effects of antibiotics and discuss promising anti-virulence targets and strategies. These targets and strategies include the bacterial type III secretion system [13,14,15], quorum sensing [16,17,18], and liposomes [19,20,21].

## 2. Downsides of Broad-Spectrum Antibiotic Use

Antibiotics have been crucial to the rise in life expectancy over the last century [22,23]. They are used to treat approximately 2.5–3 million infections per year in the US and are a hallmark of modern medicine [2]. Unfortunately, resistance to these agents is a growing problem, as 70% of infections are caused by pathogenic bacteria that contain at least one resistance gene [7]. In this section, we will review resistance as well as other downsides to the use of these agents that have also become more obvious in recent years. This includes the reduced ability to fight infections and other adverse side effects directly related to killing bacteria.

### 2.1. Resistance

#### 2.1.1. Proliferation and Mechanisms

Resistance to antimicrobial agents is not a new process. For eons, bacteria have produced secondary metabolites for self-defense purposes to aid in their survival [24]. Bacteria that produce these antibiotic compounds have naturally become resistant to them, with resistance genes often being incorporated into gene clusters with the genes for the antibiotic itself. As one example, D’costa et al. discovered 30,000-year-old bacterial DNA that encodes resistance to β-lactam, tetracycline, and glycopeptide antibiotics [24]. There is also present in the environment a number of resistance genes, even in minimally human-impacted areas [25]. This ‘intrinsic resistance’ has been proposed to make up approximately 3% of the bacterial genome [26]. The spontaneous frequency of mutations conferring resistance to an antibiotic is 10^−9^–10^−6^ dependent on species and strain, making resistance formation inevitable [27]. Now that humans have entered the evolutionary “arms race” with the clinical use of antibiotics, the prevalence of resistant bacteria is rising with the widespread use of these agents [28]. Davies and Davies have reviewed the resistance formation timeline to common FDA approved agents [29]. It is estimated that by the year 2050 that 10 million people will die from infections that can no longer be treated by available antibiotics [30].

**Figure 1:**
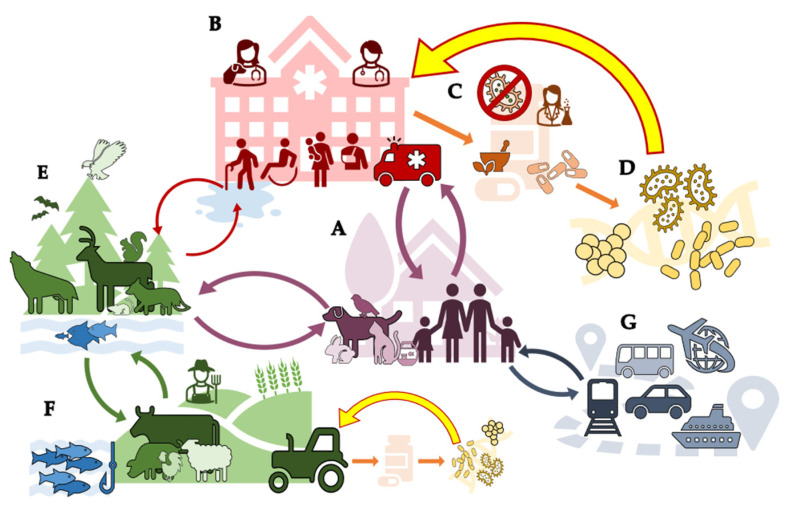
Mechanisms of resistance gene transfer and spread. (**A**) the greater community including the populace and their pets; (**B**) health care facilities including hospitals, long term care facilities, and other medical offices; (**C**) antibiotics prescribed by healthcare workers to treat infections; (**D**) bacteria mutates to become resistant to antibiotics; (**E**) wildlife serves as a reservoir for resistance genes obtained through pet interactions with animals (**A**), wastewater from health care facilities (**B**) and agriculture (**F**); (**F**) antibiotics are used as growth factors for livestock; and (**G**) travel can introduce resistance genes to communities where they were previously absent.

Along with the natural borne resistance leaking into human-populated areas and reaching pathogenic bacteria, resistance can be spread through several other mechanisms (Figure 1). This includes resistance originating from the use (and often misuse) of antibiotics to treat active infection in humans or animals such as pets or livestock, as well as antibiotic use as growth factors in agriculture. From these two origins, antibiotic resistance genes can proliferate and spread within communities and around the world. It often starts when a member of a family obtains an infection (Figure 1A) and goes to a medical care facility (Figure 1B) to obtain an antibiotic to use as a treatment (Figure 1C). The family member takes the antibiotic as prescribed, but a small number of pathogenic bacteria survive due to resistance genes (Figure 1D). The immune system can kill off these pathogens, but not before they spread their resistance genes to commensal bacteria [31]. The patient then leaves the medical facility to return home, where they spread small numbers of the newly resistant commensal bacteria with family members, including their pets (Figure 1A) [32,33,34]. The pets then interact with wildlife nearby and spread the resistance genes into the nearby forest, creating a reservoir for the genes (Figure 1E) [32,35,36]. The family also decides to take a trip overseas to visit family, thereby spreading the resistant commensals to a new country (Figure 1G) [32].

Resistance genes can also be exchanged between wildlife and livestock, meaning that they can transmit from one species to another, including the commensal bacteria of those animals [37]. Many industrial animals are reservoirs for bacteria commensal to the animal but are pathogenic to humans [38,39]. For example, pigs, chickens, and cattle are often carriers of *Salmonella* spp. and *E. coli* [40,41,42]. This pathogenic reservoir problem is worsened by the increased resistance genes found in animals. This is due to the use of antibiotics for growth promotion and prophylaxis leading to resistance genes to form at high rates among the bacterial populations within these animals (Figure 1F) [38,39]. As mentioned previously, wildlife and the environment can also serve as a reservoir for antibiotic resistance genes [39]. This can be due to interactions between companion animals with wildlife or due to pollution from health care facilities. Hospital wastewater is rife with antibiotics and their metabolites as well as bacteria. In one study by Yao et al., they found a 100% incidence rate of the antibiotic ofloxacin in the wastewater of three hospital wastewater treatment plants in China. There was also a high relative abundance of pathogenic bacteria (e.g., *Acinetobacter*, *Klebsiella*, *Aeromonas*, and *Pseudomonas*) in comparison to commensals [43]. This is especially troubling because antibiotic genes can be auto-replicative, meaning they increase over time [44,45].

Resistance to antibiotics forms even when they are used correctly, however, over-prescription and misuse can accelerate the problem. Approximately 30% of antibiotics prescribed at emergent or urgent care facilities in the US are unnecessary [46], and in the UK approximately 50% of patients with respiratory tract infections (RTIs) are treated with antibiotics even though over 70% of RTIs are viral [47]. In another study, it was discovered that some intensive care patients in South Africa were on up to 10 different antibiotics concurrently [48]. Many patients with RTIs directly request antibiotics as part of their care and physicians feel obligated to fulfill the request either to ensure patient satisfaction or to be over-cautious about the cause of infection [49].

Along with over-prescription of antibiotics, misuse of antibiotics is a large problem. Many patients do not adhere to the medication treatment as prescribed, often stopping the course after their symptoms are alleviated and discarding or saving the remaining pills. This is of particular importance when it comes to antibiotics, as storage of the unused medication often leads to self-medication at a later date when it is unnecessary, resulting in the increased emergence of bacterial resistance [50]. Approximately 28.5% of patients prescribed an antibiotic for an RTI are non-adherent to effective dosing with about 10.6% of those patients stopping the regimen before taking half of the doses given [51]. Of note, these negative trends increase with complexity and frequency of dosing. For example, of those given a three times-daily antibiotic regimen less than 10% had excellent adherence and less than 20% had acceptable adherence in comparison to 80% with excellent adherence for those given once-daily regimens [51].

**Figure 2:**
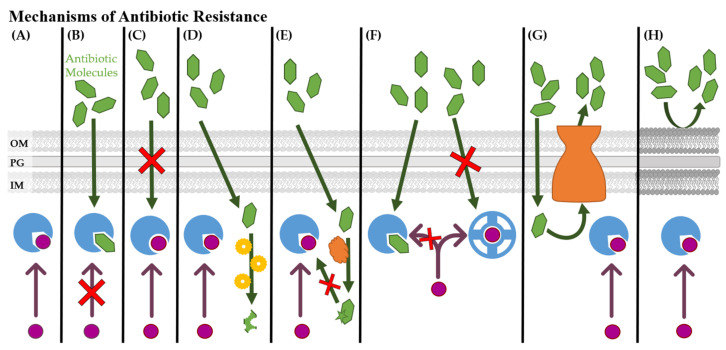
Mechanisms of antibiotic resistance. (**A**) Untreated bacterial infection; (**B**) Antibiotic treatment before resistance formation; (**C**) Mutation to the binding pocket to prevent antibiotic binding; (**D**) Recruitment of enzymes to degrade or inactivate the antibiotic; (**E**) Modification of antibiotic (e.g., methylation, acylation, phosphorylation, etc.) to prevent antibiotic binding; (**F**) Circumvention of the antibiotic target through a secondary pathway; (**G**) Upregulation of efflux pumps to remove the antibiotic; and (**H**) Decreasing cell permeability so the antibiotic does not reach MIC within the cell. Abbreviations: OM: Outer bacterial membrane; PG: Peptidoglycan; IM: Inner bacterial membrane.

The mechanism of resistance to antibiotics is highly versatile and dependent on the class of antibiotics used (Figure 2). Resistance to antibiotics is commonly thought of as point mutations to the antibiotic’s target that prevent antibiotic binding (Figure 2C). Some bacteria have evolved enzymes that inactivate the antibiotic through chemical means or physical degradation (Figure 2D) or modify the antibiotic through methylation, acylation, phosphorylation, etc. that makes it unable to bind to the active site (Figure 2E). Bacteria could also become resistant by circumventing the pathway affected by the antibiotic by utilizing a separate pathway (Figure 2F). Bacteria can also become resistant to a broad-spectrum of antibiotics by upregulating the production of efflux transporters (Figure 2G) and/or reducing permeability mechanisms (Figure 2H) [52,53,54]. Quinolones [55], tetracyclines [56,57], β-lactams [58], macrolides [59,60,61], and several other antibiotic classes are vulnerable to efflux by bacteria.

Resistance mechanisms are often used in concert to achieve multidrug resistance, and dangerous multidrug-resistant (MDR) pathogens are increasingly becoming more prevalent. *Klebsiella pneumoniae* becomes highly resistant to drugs like imipenem, ceftazidime + avibactam, and temocillin with a combination of β-lactamase production, decreased porin production and increased efflux transporter expression [62,63]. Some strains of MDR *P. aeruginosa* have become resistant to all fluoroquinolones, carbapenems, and aminoglycosides [6,64]. Although antibiotic stewardship practices such as stricter guidelines for prescriptions, prescriber education programs, and delayed prescriptions can slow the spread of resistant bacteria, the resistance problem is not going away soon [65,66]. The Centers for Disease Control and Prevention (CDC) has identified several antibiotic-resistant bacteria as “urgent” threats, including *Clostridioides difficile*, carbapenem-resistant *Acinetobacter*, drug-resistant *Neisseria gonorrhoeae*, and carbapenem-resistant *Enterobacteriaceae*. Many others like drug-resistant *Streptococcus pneumoniae* and methicillin-resistant *Staphylococcus aureus* are classified as “serious” threats.

There is, however, a cost to the bacteria associated with developing these resistances [67]. Resistant bacteria often grow slower than their antibiotic-susceptible ancestors [68]. For example, bacteria may evolve hyper-accurate ribosomes to resist ribosomal targeting antibiotics. While this leads to less antibiotic binding, and thus antibiotic activity, the ribosome functions slower, leading to an attenuated growth rate [69]. Although rare, some bacteria have even become dependent on these antibiotics for their survival [70,71,72]. For example, a strain of *P. aeruginosa* was found to be dependent on the antibiotic sulfamethoxazole. Wolter et al. showed this dependence was due to changes made in the bacteria’s membrane by sulfamethoxazole that allowed for the incorporation of an otherwise toxic phospholipid [70].

#### 2.1.2. Gene Transfer

Bacteria can undergo natural selection to evolve resistance genes when placed under selective pressure by an antimicrobial compound. This is known as vertical gene transfer (VGT). Bacteria can also transfer their genetic material, including their resistance genes, to other populations of bacteria by transformation, transduction, or conjugation [73]. This process is known as horizontal gene transfer (HGT). The use of HGT mechanisms means that a bacterial population that has never been exposed to a particular antibiotic may still become resistant to it. For example, it is believed that the gene causing vancomycin resistance in vancomycin-resistant *S. aureus* (VRSA) originally developed in *Enterococcus faecalis* and was later transferred via HGT to *S. aureus* strains [74].

**Figure 3:**
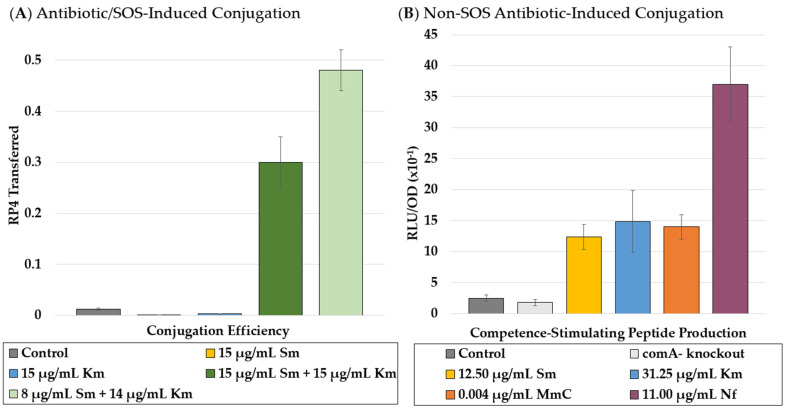
Genetic transformability/competence induced by antibiotic stress. (**A**) Antibiotic-induced SOS response-related increase in conjugative efficiency in *E. coli* after a 20 h incubation. (**B**) Luciferase activity indicating levels of CSP as controlled for by *S. pneumoniae* cell growth after 70 min in the presence of sub-therapeutic concentrations of multiple antibiotics. Abbreviations: Sm: Streptomycin; Km: Kanamycin; MmC: Mitomycin C; Nf: Norfloxacin. Data from [75,76].

Not only do antibiotics apply selective pressure, but they may also increase the rate at which mutations occur by inducing HGT, aiding bacteria in the development of subsequent antibiotic resistance (Figure 3) [75,76,77]. Antibiotics can also select for bacteria that have a higher rate of mutation, essentially training them to develop resistance faster [78]. For instance, treatment with sub-MIC fluoroquinolones and dihydrofolate reductase inhibitors promotes the transfer of plasmids conferring resistance to chloramphenicol, sulfamethoxazole, trimethoprim, and streptomycin [12]. Stress from certain antibiotics, or combinations thereof, can also induce the SOS response in some bacteria which exponentially increases conjugation efficiency [75]. Zhang et al. demonstrated this by showing the dramatic effect of certain combinations of low doses of antibiotics on the transfer of an RP4 plasmid between two *E. coli* strains (Figure 3A) [75]. More evidence of this phenomenon was observed when Prudhomme et al. showed that antibiotic stress from multiple classes of bacteria-induced genetic transformability in *Streptococcus pneumoniae*, a bacterium that does not have an SOS-response [76]. They tested four different antibiotics (mitomycin, norfloxacin, kanamycin, and streptomycin) at sub-therapeutic concentrations and found that they all induced the production competence-stimulating peptide (CSP) as monitored by luciferase assay (Figure 3B).

### 2.2. Reduced Ability to Fight Infections

One of the major downsides to antibiotic use is a reduction in the ability to fight infection after treatment, mainly due to the collateral killing of commensal bacteria. This phenomenon was demonstrated in 1950 when Terramycin proved to alter the gut microbiota in patients undergoing bowel surgery [79,80]. Antibiotics are often prescribed during hospital stays for severe infection treatment or infection prevention after surgery or in those immunocompromised. This high abundance of antibiotics and co-locality of many pathogenic bacteria within a hospital environment contributes to over 1.7 million hospital-acquired infections every year and over 100,000 deaths in the US [81]. Changes to gut microbiota due to antibiotic use, such as total volume loss and species composition ratio disruption, can last for years after the exposure [82,83] and the loss of certain commensals can be permanent [84]. One study by Roubaud-Baudron et al. indicates that early-life antibiotic exposure can increase susceptibility to infection later in life as well as increase infection severity for infections obtained during adulthood [8]. This is of particular significance as children are prescribed more antibiotics than adults [85,86].

**Figure 4:**
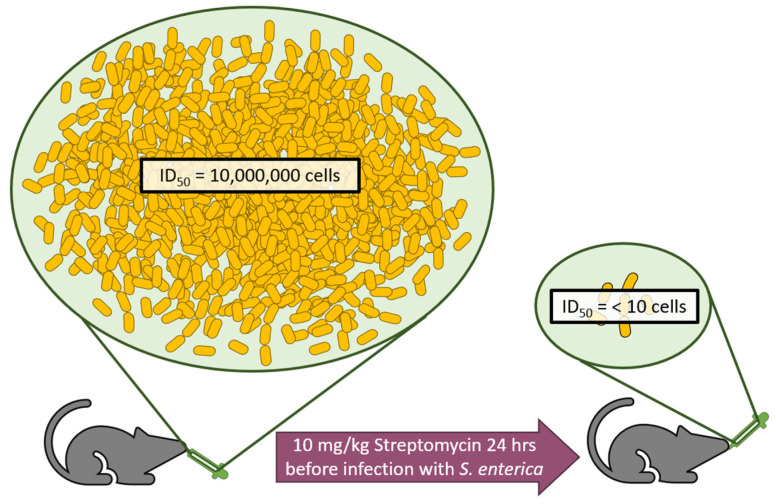
The effect of antibiotics on the ID_50_ of pathogenic bacteria. Data from [88].

The main cause for increased risk of infection after antibiotic treatment is the loss of total volume of commensal bacterial populations throughout the body, especially in the gastrointestinal tract. In normal circumstances, these commensals provide competition that keeps opportunistic pathogens in check [87]. When they are lost or significantly reduced, other species of bacteria can flourish in this niche. This phenomenon is well understood, but the extent of the problem is often not appreciated. The infectious dose of *Salmonella enterica* required to infect 50% of mice (ID_50_) is typically upwards of 10,000,000 individual cells. However, 24 h after a singular treatment of 10 mg/kg of the antibiotic streptomycin, the ID_50_ of *S. enterica* is reduced to <10 cells (Figure 4) [88]. In context, this is the difference between eating an entire improperly cooked infected chicken breast to touching a properly washed cutting board [89].

It has also been shown that acute antibiotic exposure increases the risk of traveler’s diarrhea (caused most commonly by *Salmonella* spp., *Campylobacter* spp., and enterotoxigenic *Escherichia coli*) for a year or more after treatment and multiple treatments in a short time can prevent the recovery of the microbiome indefinitely [90]. The odds of having received antibiotic treatment within 3 months before a *Salmonella* infection is twice that of baseline and is 50% higher for a year [91]. This is of particular importance because food-borne bacterial infections, such as traveler’s diarrhea, are of a higher likelihood to carry resistance genes [31]. For example, approximately one-third of the two most common species of *Campylobacter* are resistant to ciprofloxacin (28% of *C. jejuni* and 38% of *C. coli*) and a study by Koningstein et al. showed overall *Campylobacter* resistance to fluoroquinolones was 21.7% and to macrolides was 2.3% [92].

The importance of these commensals in preventing secondary infection and disease is through simple competition as well as their assistance in host metabolism [31,84]. For example, many commensal bacteria are responsible for converting primary bile acids produced in the liver (cholic acid and chenodeoxycholic acid) into secondary bile acids (deoxycholic acid, lithocholic acid, and others). These bile acids play a role in secondary infection risk, like *C. difficile* infections (CDI). Higher levels of primary bile acids correlate with higher CDI rates in patients. In comparison, healthy patients typically have more secondary bile acids than primary [93,94]. It has also been shown that bile acids have both direct and indirect antimicrobial effects on the gut microbiome composition and volume [95,96]. The majority of antibiotics are known to decrease the number of bile acid-converting bacteria in the human gut [97].

Modulation of gut bacteria is not inherently malicious to host outcomes. For example, two clinically effective drugs for treating diabetes, metformin and berberine, modulate the gut microbiota and may contribute to beneficial effects on the host. Both drugs alleviated the negative effects of high-fat diet-induced changes by reducing gut microbial diversity, but increasing short-chain fatty acid (SCFA)-producing bacteria (*Allobaculum*, *Bacteroides*, *Blautia*, *Butyricicoccus*, and *Phascolarctobacterium*) were increased [97]. It is known that increased production of SCFA’s contribute to cardiovascular health and provide anti-obesity effects [98,99]. Antibiotics such as ampicillin, on the other hand, have been shown to decrease these bacteria, which reiterates the detrimental effect they have on the human microbiome [100].

#### Case Study: *Clostridioides difficile* Infection (CDI)

In 1974, it was seen that 21% of patients treated with clindamycin (a lincosamide) developed diarrhea and 50% of those with diarrhea had pseudomembranes in their feces—a sign of CDI [101]. In the US alone, *C. difficile* cases required approximately 224,000 hospitalizations and caused at least 12,800 deaths in 2017 [102,103]. This high incidence is partly due to the significant number (estimates range from 3–26%) of people who are asymptomatic carriers of the pathogen [6]. *C. difficile* is a spore-forming bacteria, making it resistant to heat, UV, and many antibiotics. Disease symptoms, mainly caused by toxins A and B, range from diarrhea and fever in mild cases to colitis, toxic megacolon, multi-organ failure, and even death in more severe cases [104]. In total, 10–30% of patients have recurrent infections with increased risk after each occurrence [105,106], and ~20% of cases go undiagnosed due to lack of clinical suspicion or sub-optimal laboratory methods [107].

Antibiotic exposure, especially by penicillins, cephalosporins, clindamycin, and fluoroquinolones, is the highest risk determinant for CDI, with an 8–10 fold higher risk of CDI during treatment and 3-fold higher for 2 months after treatment [108,109]. Other significant factors include hospital stay length [110,111,112], age [108,113], and asymptomatic carriage [109,110,114,115,116]. CDI is becoming harder to treat over time due to a multitude of factors. Metronidazole is a 1st line treatment for CDI and currently has a clinical cure rate of ~66% [117], with >20% of infections being non-responsive [118,119]. Further exacerbating factors include increases in recurrent CDI rates [119] and the emergence of hypervirulent strains [120]. For example, the hypervirulent strain NAP1/027 represented 0.2% of all cases before 2001 [121], but now represents 51% in the US [122] and 84% in Canada [121].

Some new treatments have shown promise against CDI. Bezlotoxumab, an anti-toxin B monoclonal antibody, was FDA approved in 2016 as a combination therapy with antibiotics for the treatment of CDI [123]. Fidaxomicin is a narrow-spectrum antibiotic that was FDA approved for CDI treatment in 2011. It targets only Gram-positive bacteria, shows a low physiological effect on gut flora, and has been shown to decrease recurrent infection [124,125]. These medications are promising CDI treatments that are important developments for infection treatment, however, they do not address the root cause of the infections. Fecal microbiota transplants (FMT) have recently been used to decrease risk and treat recurrent CDI. FMT has been shown to be more effective at treating CDI than vancomycin when administered via nasoduodenal tube [126] and colonoscopically [127].

### 2.3. Adverse Side-Effects

In the US alone there were a reported 142,505 emergent care visits for drug-related adverse events that were traced to antibiotic use [128]. In addition, 20% of all hospitalized patients who received an antibiotic experienced an adverse drug event [129], and antibiotic-associated diarrhea (ADD) affects an estimated 5–39% of antibiotic users [10]. Although the majority of these can be contributed to allergic reactions (nearly 80%), a significant portion were due to other adverse effects [128]. These effects can be the result of bacterial survival tactics (often called SOS responses) induced by the antibiotic itself.

#### 2.3.1. Bacterial SOS Response Induction by Antibiotics

A prototypical example of an SOS response is the increased production of Shiga toxin by *Shigella* spp. or Shiga toxin-producing *E. coli* (STEC) in reaction to environmental factors, including antibiotic treatment [130]. Shiga toxin (Stx) is one of the most potent biological exotoxins known. A single molecule of Stx is sufficient to kill a human cell via protein synthesis inhibition and eventual apoptosis [131]. Systemic exposure to Stx causes fluid accumulation in ileal loops and renal damage in animal models and is lethal when injected directly. Humans infected with Stx-producing bacteria manifest in hemolytic uremic syndrome (HUS), a disease characterized by a triad of symptoms that all stem from the apoptotic mechanism of Stx: reduced serum platelet levels (thrombocytopenia), hemolytic anemia, and renal damage [132,133,134].

**Figure 5:**
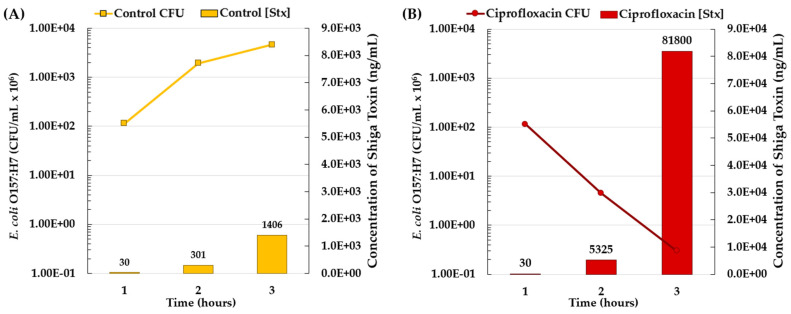
Shiga toxin induction by antibiotic use in *E. coli*. (**A**) As pathogenic *E. coli* grows there is a slight increase in Shiga toxin observed (yellow). (**B**) When ciprofloxacin is used to eliminate the bacteria, the surviving bacteria have an SOS response that includes over-production of Shiga toxin (red). Data from [135].

Although Stx is consistently produced by *Shigella* spp. and STEC, the rate of that production can be exponentially increased when the bacterial population is exposed to antibiotics that activate their SOS responses. This effect was demonstrated by Zhang et al. in *E. coli* O157:H7 in vitro (Figure 5) [135]. Three hours after 30 ng/mL ciprofloxacin addition to an *E. coli* culture there was a 17-fold increase in Stx concentration (Figure 5B). They did find, however, that not all antibiotics tested caused the SOS response (e.g., Fosfomycin). They then tested the effect of inducing bacterial SOS response has on survival in vivo.

After treatment with ciprofloxacin, there was a 3-fold decrease in fecal CFUs in comparison to the PBS control group. In contrast, mice treated with ciprofloxacin had an increase in fecal Stx in comparison to the control mice. Of note, the mice with the highest fecal Stx levels died. These mice included 67% of the ciprofloxacin-treated mice and none of the control mice [135]. Every year in the US there are approximately 17,000 shigellosis and 18,500 STEC-caused deaths [2]. The majority of these deaths occur after the patient has undergone treatment which lends to the question: Were the deaths caused by the infection or by complications induced by antibiotic treatment, such as toxins released through bacterial SOS responses?

#### 2.3.2. Endotoxin Release after Antibiotic Treatment

Exotoxins, such as Stx, are actively secreted by bacteria. Some endotoxins are only released from bacterial cells when it is disrupted or lysed, such as by antibiotics [136]. These toxins are often lipopolysaccharides (LPS) on the exterior of Gram-negative bacteria, with three distinct regions: Lipid A, an R polysaccharide, and an O polysaccharide. *Bordetella pertussis*, *Escherichia coli*, *Enterobacter* spp., *Klebsiella* spp., *Neisseria* spp., *Salmonella* spp., *Shigella* spp., *Proteus* spp., *Pseudomonas* spp., and *Vibrio cholerae* are all pathogenic endotoxin-producing bacteria [136,137]. Symptoms of endotoxin exposure range from fever, changes in white blood cell counts, disseminated intravascular coagulation, and hypotension, to shock and death. Injection of fairly small doses of endotoxin results in death in most mammals with disease progression following a regular pattern: (1) latent period; (2) physiological distress (diarrhea, prostration, shock); (3) death [136]. These toxins are also unable to be destroyed by boiling or distillation, making them very difficult to remove from wastewater and multiuse medical devices [136,138]. The FDA has a bacterial endotoxin limit of 0.5 EU/mL for medical devices and 0.25 EU/mL for sterile injectables [137], but deadly contamination still occurs [134].

Endotoxins are of particular importance when discussing antibiotics as the majority of antibiotics cause the release of these endotoxins upon bacterial cell death [136,139,140]. The Jarisch Herxheimer reaction (JHR) is specifically characterized as occurring within 24 h of antibiotic treatment of a spirochete infection and usually manifests as fever, chills, rigors, nausea and vomiting, headache, tachycardia, hypotension, hyperventilation, flushing, myalgia, and exacerbation of skin lesions [139]. Penicillins, tetracyclines, erythromycin, cephalosporins, levofloxacin, ciprofloxacin, clarithromycin, meropenem, and azithromycin can all cause JHR [139]. In sepsis, bacterial endotoxin exposure results in complications such as adult respiratory distress syndrome disseminated intravascular coagulation, and shock [141], often observed only after antimicrobial therapy has been administered [142]. In these cases, the antibiotic therapy causes a release of bacterial endotoxins and results in a Jarisch Herxheimer-like reaction [139,140].

### 2.4. Current Mediation Methods

Mediation methods, such as combination therapies, point of care and resistance susceptibility testing, narrower-spectrum therapies, and antibiotic stewardship programs have been used to mitigate issues that arise due to antibiotic use, such as resistance, reduced ability to fight infection, and adverse side effects [65,66,143,144,145,146,147]. These often help to prevent prolonged infections resulting from ineffective treatment or target specific bacterial infections to reduce side effects [148].

#### 2.4.1. Combination Therapies/Adjuvating

Combination therapy consists of using a cocktail of antibiotics and adjuvants with different mechanisms and/or spectrums of action. This is to ensure the elimination of the pathogen by overwhelming or circumventing the potential resistance mechanisms present in the patient [149,150]. Unfortunately, this can result in additional resistance gene formation in the commensal bacteria of the patient, resulting in an inability to treat infections with those antibiotics in the future [151]. These combinations are often carried out using antibiotics that are not otherwise used in treatment due to harsh side effects or toxicity risks [152]. Many of these harsher antibiotics and/or antibiotic combinations also have to be administered via IV injection, resulting in higher discomfort to the patient than if the doses could be taken orally [151,152]. This also means the time a patient must remain in a hospital is increased as they have to stay for the duration of the treatment, again raising their risk for secondary infection and mortality [110,111,112]. Another downside to combination therapy is the potential to increase the risk and severity of side effects. Allison et al. showed that using a combination therapy of piperacillin-tazobactam or vancomycin-cefepime resulted in an >150% increase in kidney disease in comparison to vancomycin monotherapy [153].

Combination therapies also include combining antibiotics with adjuvating compounds that increase the efficacy of the antibiotic through different methods. One common example of this is the use of β-lactamase inhibitors in combination with β-lactam antibiotics. Clavulanic acid, sulbactam, and tazobactam are all commonly irreversible β-lactamase inhibitors that act as suicide substrates, covalently binding to the β-lactamase active site so it is no longer functional [154]. Ceftolozane is a 5th generation cephalosporin that was FDA approved in 2014 and is paired with tazobactam for treatment [155]. Advances in this technology have also led to singular chemical moieties that can perform both antibiotic and anti-β-lactamase activities. One such example of this is Cefiderocol, which was FDA-approved in 2019 [156]. On the other hand, some clinicians are using combinations of multiple synergistic molecules to improve the efficacy of antibiotics. Recarbrio was also FDA approved in 2019 and is a combination of imipenem, cilastatin (renal dehydropeptidase inhibitor), and relebactam (β-lactamase inhibitor) [156].

Another common combination prescribed with antibiotics is prebiotics or probiotics. Prebiotics work by providing food and/or resources for the remaining commensal bacteria to help with recolonization [157]. For example, human milk oligosaccharides are known to help restore the balance between Firmicute and Bacteroidetes commensals following antibiotic therapy [158]. Probiotics are live populations of commensal bacteria that are given to patients to help recolonize the patient, resulting in a reduced risk of secondary infections and assisting in out-competing pathogenic bacteria [159,160]. There is debate as to the efficacy of probiotics due to the high degree of variability in their administration. However, a meta-analysis of probiotic use with antibiotics to treat acute diarrhea in children “supports the potential beneficial roles of probiotics and symbiotics for acute diarrhea in children” [161]. At least two independent studies have shown the use of *Lactobacillus rhamnosus* GG was able to successfully decolonize patients with vancomycin-resistant enterococci [162,163]. Prebiotics and probiotics have also shown additive effects when used in concert [164]. These types of treatments are often helpful in reducing the risk of secondary infection, especially when treating gastrointestinal infections, but once again do not completely eliminate the problem [159,160].

#### 2.4.2. Point of Care and Resistance Susceptibility Testing

Point of care testing (POCT) and resistance susceptibility testing are other strategies that have grown in popularity as a method of mediating the side effects of antibiotic use. In this review, a medical test is considered point of care if it meets two criteria: it is performed near where the patient is being seen and it takes 15 min or less to obtain results. They are often carried out in a manner that allows a patient to be examined, tested, and prescribed within the same visit [165]. These methods help clinicians to decide the specific treatment regimens by elucidating the specific pathogen and/or any resistances that the pathogen may be harboring [166]. Widespread access to POCT can reduce the number of incorrect diagnoses and therefore misprescribed antibiotics. For example, access to a POC malaria rapid diagnostic test in Zambia led to a four-fold reduction in inappropriate antimalarial prescribing [48]. The use of POCT in primary care has increased by 45% from 2004 to 2013 and has continued to grow in popularity [167].

Many hospitals only offer POCT or resistance susceptibility testing after a patient has shown negative progress during a first-line treatment due to the costs associated with the time, personnel, and equipment associated with performing these tests [165]. This also means that there is a disparity in the clientele that can have the tests performed, leading to a high degree of socioeconomic discrepancy in access [165,167,168,169]. Weihser and Giles showed that an increase in the use of POCT in ambulatory care, in essence, an earlier timepoint, reduces the overall number of patient bed days spent in the hospital. This overall reduces the financial and workload burden on the health care facility. These reductions lead to better overall health outcomes as patients with severe disease were not delayed in their transfer to ICU or other relevant units [170].

There is a significant problem of quality control in terms of the testing itself. These tests are often performed by in-house laboratory staff and/or by an external laboratory that is closely associated with the health care facility in question. These facilities and tests are not regulated to the same degree that medications or medical devices are. This leads to a lacking of safeguards for their accuracy and efficacy [171]. For example, in 2018 Saraswati et al. found significant heterogeneity in POCT for the diagnosis of scrub typhus, a disease caused by the bacterium *Orientia tsutsugamushi*. There was a pooled sensitivity rate of 66.0% due to the wide array of methodology and variation in quality of the tests offered to patients [172]. The same quality control issues are seen in viral focused POCT as well. Mak et al. reported the sensitivity of a SARS-CoV-2 rapid diagnostic test was potentially as low as 11.1%, even though the manufacturer had claimed it to be 98% [173]. This lack of quality control and regulation leads to potentially thousands of false-negative tests and increased spread of disease [171].

#### 2.4.3. Narrow Spectrum Treatments

Narrow-spectrum antibiotics have a lower complication risk as they target only a subset of bacteria [143]. Narrow spectrum antibiotics are subject to much lower levels of selective pressure because they target specific bacterium [143,144,145,146]. In theory, this will decrease the speed of resistance formation and proliferation [145]. They also help to prevent disease by keeping more human commensal flora intact in comparison to broad-spectrum antibiotics [146]. This targeted approach can allow for higher doses to be used which may reduce the treatment time [143]. They also often require diagnostic testing to determine bacterial species, and occasionally even strain. This can lead to an increased total time the patient has an infection before receiving treatment [144].

Bacteriophages are viruses that selectively target and infect bacteria. When employed as therapeutics, they work similarly to narrow-spectrum antibiotics as they can be genetically modified to be host-specific. Bacteriophages are viruses at their core, allowing them to multiply inside the bacteria and be released upon lysis. This can result in an increasing number of phages over time, a vital phenomenon not observed in other antimicrobial treatments [174]. Studies have demonstrated phage therapy effectively treats infections caused by resistant bacteria such as *Listeria monocytogenes*, *Campylobacter jejuni*, and *Salmonella* spp. In 2006, the FDA approved the use of a combination of six phages to be sprayed on ready-to-eat meat and poultry to eliminate *L. monocytogenes* [175]. Bacteriophages can have similar effects to prophylactic antibiotic use and have been explored as replacements for antibiotics that are used as growth factors in livestock production [176,177,178]. Quality control and standardization of bacteriophage therapies have been a persistent and difficult challenge. The high specificity of bacteriophages makes them not suitable for patients with multiple infections or those with infections unable to be identified by diagnostic testing. In addition, phage resistance emerges quickly, necessitating the use of a cocktail of phages to slow resistance formation [177]. Bacterial exo- and endotoxins can be encoded by bacteriophages for various reasons [179]. These genes can be transferred to commensal bacteriophages and bacteria, leading to a potential for metabolic endotoxemia [180].

#### 2.4.4. Antibiotic Stewardship Case Study: Choose Wisely Canada™

Antibiotic stewardship programs that have become a common way that governments can distribute information and recommendations regarding antibiotic use. This includes the Choose Wisely^®^ program in the United States [181], Start Smart—Then, Focus in the United Kingdom [182], and national action plans in other countries [183,184,185,186,187,188]. In 2014, Canada implemented an initiative, called Choose Wisely Canada™, that endeavors to reduce unnecessary testing and treatments in healthcare [189]. Included in this initiative is a campaign to use antibiotics more sparsely and/or only when necessary. As such this program’s successes and difficulties will be examined as a case study for antibiotic stewardship programs. There are over 15 recommendations developed by Canadian national clinician societies that encourage the judicious use of antibiotics that fall into five major categories: 1. Reduce antibiotics for urinary tract infections (UTI) in older people; 2. Treating sinus infections: Don’t rush to antibiotics; 3. Colds, flu, and other respiratory illnesses: Don’t rush to antibiotics; 4. Preventing infections in the hospital: Watch out for these two practices; and 5. Sometimes no antibiotic is the best prescription [190].

Approximately 50% of older adults have bacteria in their urine that is not considered a contributing factor to UTIs [191]. This results in a high incidence of misdiagnosis due to common diagnosis testing for UTIs, such as dipstick testing and urine culture [191,192,193]. These techniques test for the presence, or lack, of bacteria and do not discriminate between pathogenic or commensal/mutualistic bacteria [194]. Many patients with positive dipstick tests will not exhibit symptoms of infection, such as fever, painful urination, dysuria, or urination frequency changes [191]. The Use Antibiotics Wisely campaign suggests refraining from the administration of dipstick, urine culture, or urinalysis tests as a means of UTI diagnosis in older adults. Instead they suggest observing symptoms and treating as they appear, not preemptively when bacteria is found in urine [190].

Up to 50% of antibiotics prescribed for respiratory tract and sinus infections in non-hospital settings are unnecessary [46,47]. Most respiratory, sinus, and ear infections are caused by a virus and as such an antibiotic will not be effective as a treatment, yet patients often insist on a prescription even when not required [195,196]. To combat this, Choose Wisely Canada™ is suggesting spending more time educating patients on the potential side effects of antibiotic use, such as stomach problems, dizziness, or rashes, to discourage patients from insisting on antibiotic use as well as prescribing delayed prescriptions that can reduce overall numbers of prescriptions filled [190,197]. A delayed prescription is a prescription that falls into the following categories: 1. Re-contact—the practice by phone to request a prescription; 2. Post-dated prescription; 3. Collection—placement of prescription at reception; and 4. Patient-led—giving the patient a prescription with advice to delay. Approximately 30% of patients given a delayed prescription fill it as opposed to nearly 90% of patients given a non-delayed prescription [198,199].

Hospitals are one of the most common places where overprescription and overtreatment occur and are a breeding ground for superbugs [81]. Choose Wisely Canada™ has pointed out two main over-used medical practices that can increase the risk of infection in hospitals to increase public awareness: urinary catheters and ulcer drugs [190]. The risk of infection increases significantly after a urinary catheter is in place for two days. They are commonly used after surgery but are often left in longer than necessary for the convenience of staff. Ulcer drugs, such as proton-pump inhibitors and histamine H2 receptor antagonists, are used to prevent stress ulcers and gastrointestinal bleeding, but up to 75% of patients prescribed these drugs after a hospital stay do not need them [200]. These drugs can kill off commensal bacteria, thus increasing the risk for opportunistic pathogenic infections. One study showed that patients on ulcer drugs are approximately twice as likely to obtain *C. difficile* infections [200].

The success of these programs is difficult and complex to measure. A study by Tannenbaum et al. showed that after an educational intervention that was part of the Choose Wisely Canada™ initiative 62% of patients initiated a conversation with their physician or pharmacist regarding the medication in question. This resulted in 27% of them changing their care plans, as opposed to 5% of patients who did not receive the intervention [201]. Another study found a 41% decrease in overall laboratory tests since the program start, resulting in an estimated $215,000 in savings while maintaining the quality of care [202]. Unfortunately, the cost-saving aspect of the program has garnered some criticism, as some view the initiative as a cost-cutting method at the expense of the patient, undermining the physician-patient relationship and trust [203]. Others have criticized the initiative for being too simplistic and only emphasizing “agreed-upon, well-established practices” [204], or claim the multiple guidelines for each topic will result in burnout and confuse physicians as to which protocol to follow [205]. The overall goal of this initiative has been effective at reducing “just in case” testing and treatment but its long-term impact on mitigating the downsides associated with antibiotic use has yet to be seen.

#### 2.4.5. Infection Prevention Measures Case Study: COVID-19 Pandemic

Preventing the spread of infectious disease would reduce the overall need for antibiotics, thereby reducing antibiotic over-prescription and misuse. During the COVID-19 pandemic infection prevention measures, such as mask mandates, emphasizing hand washing, disinfection, and social distancing, were put in place to prevent the spread of SARS-CoV-2 [206,207]. These precautions resulted in an overall reduction in community spread of other respiratory diseases during the 2020 season in comparison to 2019, including influenza (17.4% decrease), enterovirus (51.6% decrease), and all-cause pneumonia (18.8% decrease) [207]. Despite the additional training of medical staff on infection control measures, there was no decrease in nosocomial infection rates. One example of this is carbapenem-resistant *Enterobacteriaceae* (CRE); in 2019 the infection rate of CRE in ICUs was 6.7%, but by April 2020 the rate was at 50% [208]. This demonstrates that even in the hyper-aware environment of a global pandemic serious bacterial infections are still prevalent and have the potential to grow in number affected. It should also be noted that funding and publishing research on infectious diseases other than COVID-19 during this time has been significantly reduced and will have serious impacts on the future of the field (reviewed in [209,210,211,212]).

## 3. The Case for Anti-Virulence Therapeutics

Traditional antibiotics work by disrupting essential cellular functions of bacteria. A major downside to this approach is that these cellular functions are widely conserved and present in both pathogenic and commensal bacteria. In contrast, anti-virulence therapies target mechanisms in bacteria that are essential for pathogenesis but are not essential for cell viability [144,213]. A major difference between antibiotics and anti-virulence therapies is that the latter does not directly cause selective pressure. This should, in theory, reduce the rate at which resistance to anti-virulence therapies occurs [145]. Collectively, anti-virulence therapies allow the host’s natural immune response to eliminate the pathogen rather than killing a pathogen outright [214,215,216,217]. A major advantage to this approach is that bacterial SOS responses have yet to be implicated as a result of treatment [218].

The bacterial type III secretion system (T3SS) is a virulence factor used by most pathogenic Gram-negative bacteria to cause infection by injecting virulence proteins, called effectors, that reprogram the host cell machinery and allow evasion of the host immune response. The T3SS is absent from commensal bacteria and as such, any therapies targeting it should not affect commensal bacteria [15]. Some virulence factors, like the T3SS, are regulated by quorum sensing (QS), a grouping of pathways that regulate gene expression in a concentration-dependent manner. QS is used by both pathogenic and commensal bacteria. Compounds inhibiting QS may not be lethal to bacteria but could prevent colonization and infection by reducing the infectivity of pathogens. In recent years, the use of liposomes has emerged as an anti-infective strategy for treating infections caused by pathogenic Gram-positive bacteria. This strategy is not limited to one specific pathogenesis pathway, and the merits of this approach will be discussed in this section. These examples will be used to demonstrate the potential of direct ani-virulence targets (T3SS), anti-virulence signaling targets (QS), and indirect anti-virulence strategies (liposomes).

### 3.1. The Bacterial Type III Secretion System (T3SS)

The type III secretion system (T3SS) is used by many pathogenic Gram-negative bacteria to cause and maintain an infection. Pathogens using a T3SS include *Chlamydia trachomatis*, *Escherichia coli*, *Pseudomonas aeruginosa*, *Salmonella enterica*, *Shigella* spp., *Vibrio cholerae*, and *Yersinia pestis* [219,220]. The T3SS is highly conserved between bacterial pathogens of a particular genus [221]. This conservation can result in high recognition amongst different species or serovars of bacteria. The T3SS is a syringe-like apparatus that translocates effector proteins directly into a host cell [220,222]. These effectors hijack the host cell machinery to allow for colonization and to subvert the host immune response. These mechanisms include interference with actin and tubulin, gene expression, cell cycle progression, or induce programmed cell death in their host [223]. Bacteria with a nonfunctional T3SS have attenuated virulence but are still capable of growth, making them a perfect target for anti-virulence therapeutics [222]. This also lends to the theory that T3SS inhibition will reduce selective pressure on the pathogen, resulting in slower resistance formation to T3SS inhibitors [219]. The T3SS is specific to pathogens, meaning any interventions targeting it should not affect commensal bacteria [224]. Although no T3SS inhibitors have been FDA approved, the T3SS is one of the most validated anti-virulence targets, with many compounds in development (Table 1) [225].

**Figure 6:**
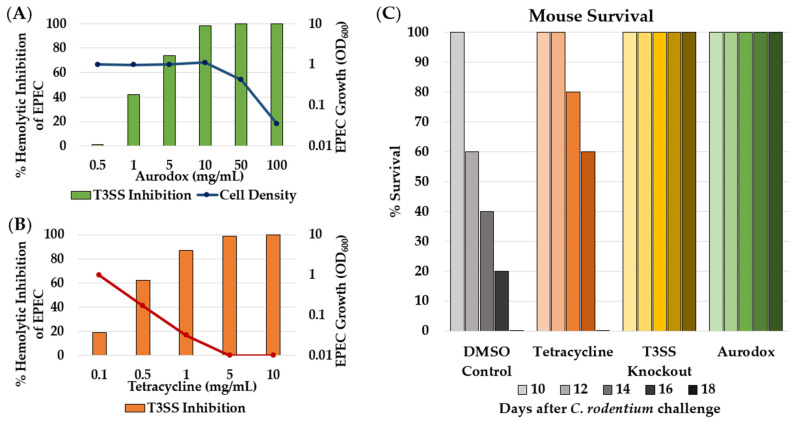
In vitro and in vivo activity of T3SS inhibitor aurodox. (**A**) Concentration of up to 10 mg/mL aurodox dose-dependently inhibit the T3SS without effecting cell growth; (**B**) The decrease in hemolytic activity is due to tetracycline killing bacteria; (**C**) 100% of mice given aurodox survived to day 18 after infection, while none treated with tetracycline survived. Data from [226].

Small molecule inhibitors of the T3SS have been shown to increase survival rates after infection with otherwise lethal doses of the bacterial pathogen (Table 1) [14,232] McHugh et al. showed the ability of the natural product aurodox to decrease effector protein secretion and decrease the infectious potential of enterohemorrhagic *E. coli* (EHEC). Transcriptomal analysis of genes affected by aurodox showed downregulation of 25 of the 41 genes related to the T3SS, including ler, a major activator of the T3SS [228]. This suggests aurodox acts as a gene repressor and not by directly binding to T3SS, although the true mode of action is still unknown. Aurodox was shown to prevent T3SS mediated hemolysis, with an ID_50_ of 1.5 μg/mL (Figure 6A) [227,228]. Kimura et al. collected further data to analyze the effectiveness of aurodox on alleviating T3SS-mediated infection using an in vivo mouse model [226]. Mice were infected with *Citrobacter rodentium*, a murine variant of enteropathogenic *E. coli* (EPEC), and then either treated with 10% DMSO as a control, a single dose of tetracycline (200 mg/kg), or aurodox (25 mg/kg) every 24 h for four days. All mice treated with aurodox or a functional T3SS knockout strain survived, while those treated with tetracycline did not (Figure 6C). In addition, treatment with aurodox does not induce Shiga toxin production in EHEC, suggesting promise for the use of T3SS inhibitors to treat infection [218].

Therapeutics targeting the needle tip protein of the T3SS have gone into clinical trials. One of these, termed KB001-A, is a human PEGylated IgG monoclonal anti-PcrV Fab that is proposed to form a secretion blockade mechanism of pathogenesis prevention for *P. aeruginosa* [233,234,235,236,237,238,239]. KB001-A has undergone phase I and II clinical trials for both ventilator-associated *P. aeruginosa* and treatment of chronic pneumonia in cystic fibrosis patients but did not advance to phase III trials due to a lack of efficacy [235,236,240]. More recently MEDI3902, another anti-PcrV mAb, has entered human clinical trials. This bispecific antibody targets both PcrV and Psl exopolysaccharide, an anti-biofilm formation target. MEDI3902 was shown to dose-dependently increase survival, reduce lung inflammation, and decrease bacterial loads in both rabbit and mouse *P. aeruginosa* challenge models [241]. Le et al. showed MEDI3902 was effective as a treatment and a prophylactic for acute blood and acute lung *P. aeruginosa* infections [242]. MEDI3902 performed well in phase I clinical trials in the US [229,230]. Although a single dose of MEDI3902 was shown to provide good pharmacokinetics and pharmacodynamics in phase II trials, it did not achieve primary efficacy [231]. The results from anti-T3SS therapies are promising and these examples are just the beginning.

### 3.2. Quorum Sensing

Quorum sensing (QS) is the process used by bacteria to control gene expression in response to cell density and external factors sensing [16,17,18]. Small molecules, called autoinducers, are produced by bacteria and release to be sensed by other bacteria. These allow a bacterial population to sense neighboring cells sensing [16,17,18]. QS is used to control a variety of critical functions related to growth, colonization, and pathogenesis. These including biofilm formation, virulence factor deployment, and antibiotic resistance gene expression [243]. Inhibition of QS could prevent pathogenic bacteria from coordinating virulence factor usage sensing [16,17,18]. This could reduce the pathogen’s infectivity, allowing the host immune system to clear the infection more effectively. QS inhibition could also increase the efficacy of existing antibiotics by virtue of inhibiting antibiotic resistance gene expression sensing [16,17,18]. This could allow existing antibiotics to have increased time as first-line therapies. The use of QS inhibitors as anti-virulence therapeutics is a growing field with many promising compounds (Table 2).

There are many different interconnected mechanisms controlled by QS, so a highly simplified explanation of the moderately well described QS pathways of *Pseudomonas* spp. will be used as an example [16,243]. In this section we discuss two groups of signaling molecules, quinolones and acyl-homoserine lactones (AHLs), that control three pathways of the *Pseudomonas* QS system: *Pseudomonas* quinolone signal (PQS), *las* signaling, and *rhl* signaling. PQS is induced by quinolone accumulation and results in increased cell–cell signaling, virulence protein expression (e.g., the T3SS), iron acquisition, oxidative stress, antioxidative response, and modulation of host immune responses. AHLs induce both *las* and *rhl* signaling. *las* upregulates both PQS and *rhl* QS pathways, as well as biofilm formation and other virulence factors. *rhl* signaling induces the production of toxins such as rhamnolipids, pyocyanin, and hydrogen cyanide [243]. These pathways are all interconnected in a highly complex manner that has been left out of this review for brevity. More in-depth reviews of QS systems, including *Pseudomonas* QS, are referenced in Table 2.

One of the many natural products identified with QS inhibitory activity is coumarin, the parent compound of a class of plant phenolics. Coumarin was shown to decrease the expression of multiple QS genes in *P. aeruginosa* strain PA14, including *pqsA* and *rhlI*, as well as decrease virulence phenotypes such as swarming motility and phenazine production [246]. Coumarin was found to inhibit PA14 biofilm formation, a mechanism mainly controlled through las signaling [246]. This indicated coumarin had a role in all three major *Pseudomonas* QS pathways. The biofilm inhibitory capabilities of coumarin was shown in other Gram-negative and Gram-positive bacteria, including *E. coli*, *Edwardsiella tarda*, *Vibrio anguillarum*, and *S. aureus*, thus showing the broad spectrum of anti-QS activity of coumarin [246,259].

Approximately 65% of infections are caused by biofilm-forming bacteria [260], and these biofilms are regulated by QS [261]. Biofilms are an immobile community of bacteria living on a surface that share resources within an extracellular matrix [261]. Biofilms protect from sudden changes in pH, osmolarity, nutrients scarcity, mechanical, and shear forces [262,263]. They also prevent antibiotics and host immune cells from accessing bacteria within the biofilm community [264,265]. Methods for the inhibition of biofilms include deploying compounds that disrupt or remove established biofilms and the use of antibiotics, antimicrobials, or antibiofilm compounds on a matrix to disrupt biofilm formation [260].

Furanone A, a natural product isolated from the algae *Delisea pulchra*, has been shown to inhibit QS through *las* modulation. A derivative of Furanone A, named C-30, was found to interfere with biofilm formation in both planktonic cultures and established biofilm colonies of *P. aeruginosa* [220]. It was later shown to have synergistic effects when combined with an antibiotic, increasing the effectiveness of the antibiotic tobramycin, despite having no antibiotic activity itself. Although not quantified, qualitative data of colonies treated with the combination showed a drastic decrease in biofilm matrix and increase in cell death by two to three magnitudes, with only 5–10% of cells remaining alive after treatment [220]. This was due to the antibiofilm activity of C-30 which allowed tobramycin to reach the bacteria more easily. It also likely contributed to a decrease in antibiotic resistance gene expression, thereby increasing the efficacy of tobramycin. This example sets the precedent that anti-biofilm and/or anti-QS compounds can be used in conjunction with known antibiotics to increase their efficacy and lifespan as therapeutics.

### 3.3. Liposomes

**Figure 7:**
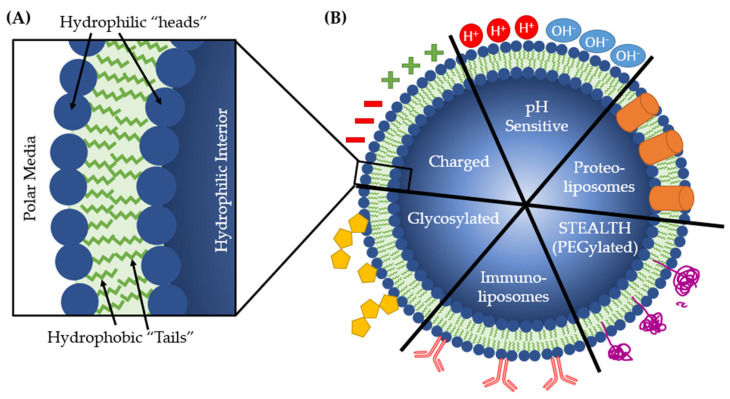
Structure and type of liposomes. (**A**) Liposomes are composed of phospholipids containing hydrophilic “heads” and hydrophobic “tails” that organize into micelles. Within the micelle is a hydrophilic interior while the exterior is polar media; (**B**) Modifications to liposomes can greatly enhance the ability of liposomes to localize to a desired target. This includes incorporating charged or pH-sensitive moieties, PEG, or carbohydrate components into the phospholipid bilayer or coating liposomes with proteins or immunoglobulin.

Liposomes are cell-like vesicles formed by an agglomerate of phospholipids. Phospholipids are amphipathic due to a hydrophobic “tail” and a hydrophilic “head” (Figure 7) [19,20,21]. Because of this, they can spontaneously form liposomes when placed into polar, typically aqueous, media. The phospholipids arrange into a bilayer, like a cell membrane, enclosing a sphere of the media (Figure 7) [266]. Liposomes can be synthesized in a laboratory using a variety of lipids, cholesterol, and lipoproteins, making them highly customizable [267]. Liposomes have applications in many different fields: cosmetics, medical imaging, vaccination, and drug delivery [268].

The use of liposomes can increase the local concentration of drugs at the therapeutic site. This can improve the efficiency of therapies while simultaneously decreasing off-target toxicity. Liposomes can also improve the pharmacokinetics and biodistribution of a delivered drug, enhancing drug absorption to increase plasma concentration, and increasing the half-life of drugs [269,270]. Variations in the type and proportions of components used to form liposomes, as well as the method used to form them, can give them a range of pH sensitivities, sizes, number of bilayers (called lamellae), temperature sensitivities, and membrane fluidities [267]. Because of their customizable nature, liposomes have been used for delivering antifungals [271,272], antineoplastics [269,273,274,275], antibiotics [276,277], and even gene- or drug-therapies for Alzheimer’s disease [278,279].

Because liposomes can target a specific area, they can allow for lower toxicity than their free-drug counterparts [269,271,272,275,280]. Bakker-Woudenberg et al. showed free ciprofloxacin can cause toxicity at a concentration of 40 mg/kg/dose but is tolerated to 160 mg/kg/dose when delivered with liposomes [280]. Liposomes can also increase the general activity of the drug they carry [277,280]. For example, using cationic liposomes to deliver clarithromycin reduced the minimum bactericidal concentration (MBC) of clarithromycin to 16 mg/L against several highly resistant clinical strains of *P. aeruginosa*, compared with 512 mg/L for free drug and 64 mg/L for anionic liposomes alone [277].

More recently, liposomes have been designed for anti-virulence therapies [281]. Gram-positive bacteria, such as *Staphylococcus* spp. and *Streptococcus* spp., interact with eukaryotic host cells to deliver virulence factors across the cell membrane [282] or release toxins into the surrounding media [283]. Liposomes can be designed as eukaryotic host cell mimics that act as bait for pathogens or as toxin scavengers. This can result in pathogens. To unproductively use their virulence factors and resources on the liposome instead of eukaryotic host cells. This ultimately lowers the infectivity of the pathogen, which in turn increases the minimum infectious dose required to cause infection. Equally important is that anti-virulent liposomes have broad-spectrum pathogenesis-hindering activity. An anti-virulent liposome injection was able to save mice from an otherwise lethal dose of *S. pneumoniae* or *S. aureus* if given, at most, 10 h after infection [281]. This means one formulation of liposomes could be used to treat a variety of infections similarly to broad-spectrum antibiotics but without the harmful side effects.

**Figure 8:**
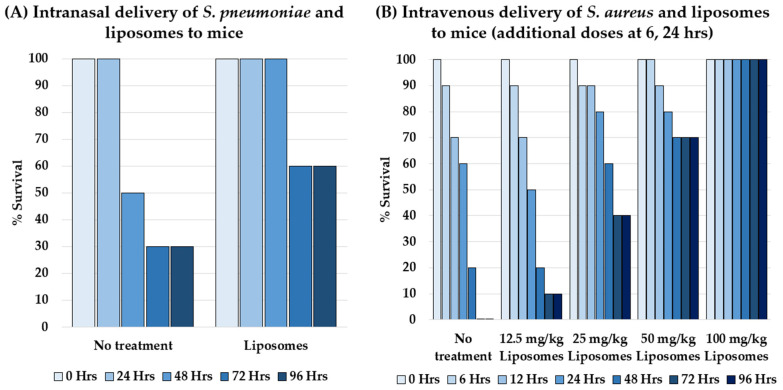
Treatment of *S. pneumoniae* and MRSA with liposomes. (**A**) Treatment with 100 mg/kg of a 1:1:1:1 mixture of Ch:Sm (66 mol % Ch) + Ch:PC (66 mol % Ch) + Sm + Sm:PC (75 mol % Sm) liposomes was able to double the survival rate of mice infected with *S. pneumoniae*. (**B**) Liposomes dose-dependently increase the survival rate of mice infected with MRSA, with 100 mg/kg and higher concentrations of a 1:1 mixture of Ch:Sm + Sm liposomes resulting in 100% survival.

Many bacterial toxins bind to membrane components that are host-specific, such as cholesterol (Ch), phosphatidylcholine (PC), or sphingomyelin (Sm) [281]. Pathogen-released pore-forming toxins such as streptolysin O (SLO) bind to Ch or PC and then self-associate with other SLO monomers to form a ring- or arc-shaped complex that creates lesions in the host cell membrane, reducing the integrity of host cells [284]. Liposomes containing large proportions of the pathogen-targeted membrane components can be more attractive for toxins than a real cell. Henry et al. found that toxin-sequestering liposomes are effective in vivo by reducing bacterial load in the blood and lungs and doubling the survival rate of mice intranasally infected by *S. pneumoniae* (Figure 8A) [281].

Liposomal cholesterol is a key target for many pathogens. Liposomes composed of the largest proportion of Ch possible (66 mol %) along with either PC or Sm were the most effective for neutralizing an array of toxins [281]. In some instances, however, a combination of differently formulated liposomes may be required for maximum neutralization of toxins. Henry et al. showed that liposomes composed of Ch:Sm or Ch:PC protected monocytes in vitro from MRSA only partially, whereas a combination of both Ch:PC and Sm liposomes led to 100% survival of the monocytes (Figure 8B) [281]. This suggests that MRSA secretes at least two toxins: one that is neutralized by Ch:PC liposomes and one that is neutralized by Sm liposomes.

Using liposomes to prevent toxins from damaging cells has the secondary benefit of preventing the cytokine storm and septic shock associated with an inflammatory response [281]. This is a major advantage of anti-virulence liposomes over antibiotics; pathogen lysis by antibiotics can cause a very sudden release of toxins and strongly activate the destructive inflammatory response whereas liposomes sequester the pathogen or toxins [285]. For example, the pathogen *S. pneumoniae* carries endotoxins that are released only when the cell is lysed [286]. This toxin flood release may be counteracted by combination therapy of liposomes and antibiotics, which was shown to increase host survival chances beyond that of antibiotics alone, as liposomes can neutralize the toxins released by bactericidal agents [281].

### 3.4. Combination Therapies

One disadvantage of using anti-virulence therapies is that by design they do not actively clear infectious bacteria from a patient. This can be problematic for patients who are immunocompromised, either naturally or through the severity of infection. Notwithstanding the potential lack of efficacy, these therapies could be used in combination with traditional antibiotics to reduce the adverse effects of using antibiotics alone. These combination therapies may also allow for reduced drug dosages and treatment duration, leading to reduced resistance formation and side effects [73].

Evidence of this synergistic approach was demonstrated by Secher et al. in a study of an anti-*P. aeruginosa* mAb targeting the needle tip of the T3SS with the broad-spectrum antibiotic meropenem. An additive effect was observed when the combination was given to patients. Meropenem-resistant infections had similar efficacy to treatments with the mAb alone against meropenem-sensitive infections, showing an ability of combination therapy to overcome drug resistance [287]. Le et al. demonstrated that MEDI3902, another anti-*P. aeruginosa* mAb targeting the T3SS, showed enhanced activity against *P. aeruginosa* infections when administered in combination with a subtherapeutic dose of meropenem [241]. Anti-T3SS mAb therapy had increased effectivity when administered in combination therapy with either ciprofloxacin, tobramycin, and ceftazidime against acute *P. aeruginosa* infection [288]. These results show that regardless of their success as individual agents, anti-virulence therapeutics can also be employed in combination with antibiotics to reduce the overall downsides to antibiotic use by reducing doses and time of treatment.

## 4. Conclusions

The use of antibiotic therapies for the treatment of bacterial infections has been indispensable during the rise of modern medicine. However, their use does not come without significant downsides. Anti-microbial resistance gene formation and proliferation is the most notorious of these and has rendered many antibiotic agents ineffective. Many antibiotics reduce the ability of patients to fight infection after treatment due to the loss of commensals. Antibiotics may also cause the induction of bacterial SOS responses that can result in the release of toxins which lead to serious side effects. To mediate these downsides, clinicians have tried to implement multiple mediation methods or alternative care plans. These include the use of combination therapies, point of care testing, narrow-spectrum treatments, antibiotic stewardship programs, and increased infection control. Unfortunately, these mediation methods serve only to reduce and slow the problem, not eradicate the cause.

Anti-virulence therapies have been proposed as one solution to the downsides of antibiotic use. Anti-virulence agents do not directly cause bacterial cell death, instead, they target mechanisms used by pathogens to cause infection and evade the host immune response. These agents exhibit reduced selective pressure compared to traditional antibiotics, thereby mitigating resistance formation. In contrast to antibiotics, anti-virulence therapies do not affect commensal bacteria that protect against secondary infections. Additionally, anti-virulence agents do not induce bacterial SOS-responses that are often responsible for severe side effects associated with antibiotic treatment. We have described three groups of promising anti-virulence targets and therapies: the T3SS, quorum sensing, and liposomes. These therapies have successfully demonstrated the potential for development into bacterial infection treatments either alone or in combination with antibiotics.

## Figures and Tables

**Table 1:** Select anti-T3SS therapies in development.

Anti-Virulence Compound	Mode of Action	Bacteria	Stage
Aurodox [226,227,228]	Downregulation of T3SS-related genes	EPEC and EHEC	Animal Models
Compound D [14]	Secretion blockade	*Yersinia* and *Pseudomonas* spp.	In Vitro
Compounds 7146 and 1504 [14]	ATPase inhibition	*Yersinia* and *Burkholderia* spp.	In Vitro
INP175 [14]	ATPase inhibition	*P. aeruginosa*, *Y. pseudotuberculosis*, and *C. trachomatis*	In Vitro
MBX 23 [14]	Needle subunit inhibition	*Pseudomonas*, *Chlamydia*, and *Yersinia* spp.	Animal Models
Anti-PcrV monoclonal antibody (MEDI3902) [14]	Needle tip inhibition	*P. aeruginosa*	Clinical Trials [229,230,231]
Salicylidene acylhydrazides (e.g., MED055, RCZ12, and INP040, etc.) [14]	T3SS formation inhibition	*Yersinia*, *Chlamydia*, *Salmonella*, *Shigella*, EHEC, *Xanthomonas*, and *Erwinia* spp.	Animal Models
Thymol [232]	Translocation inhibition	*Salmonella* spp.	Animal Models
Anti-Tir antibody (TD4) [14]	Adhesion inhibition	EPEC and EHEC	Animal Models
2-imino-5-arylidene thiazolidinone [14]	Basal body alkaline phosphatase inhibition	*Salmonella*, *Pseudomonas*, and *Yersinia* spp.	In Vitro
12(4,6) and 12(6,4) [14]	Needle subunit chaperone inhibition	*Pseudomonas* spp.	Animal Models

**Table 2:** Select anti-QS therapies in development.

Anti-Virulence Compound	Mode of Action	Bacterium (QS Pathway Reviews)	Stage
Azithromycin [244]	Decrease QS gene expression	*Pseudomonas* spp. [245]	Clinical Trials
Furanone derivatives (e.g., C-30) [220]	*las* inhibition	*Pseudomonas* spp. [245]	In Vitro
Coumarin [246]	Decrease QS gene expression	*E. coli* [247], *P. aeruginosa* [245], *S. aureus* [248], and *Vibrio* spp. [249]	In Vitro
Cyclic dipeptides [250,251]	Indicated in reporter assays	*E. coli* [247], *P. aeruginosa* [245], and *Vibrio* spp. [249]	In Vitro
DPD derivatives [17]	LrsK inhibition	*Salmonella* spp. [252]	In Vitro
Epigallocatechin-3-gallate [253,254]	Decrease QS gene expression	EHEC [247], *Pseudomonas* [245], *Salmonella* [252], and *Staphylococcus* spp. [248]	In Vitro
Hamamelitannin [255]	Peptidoglycan biosynthesis and eDNA release inhibition	*Staphylococcus* spp. [248]	Animal Models
LED209 [256]	QseC inhibition	EHEC [247] and *Salmonella* spp. [252]	Animal Models
Sinefungin [257]	Inhibition of AI-2 Synthesis	*Streptococcus* spp. [258]	Animal Models
